# Stochastic mean-field formulation of the dynamics of diluted neural networks

**DOI:** 10.1186/1471-2202-16-S1-P263

**Published:** 2015-12-18

**Authors:** David Angulo-Garcia, Alessandro Torcini

**Affiliations:** 1 Istituto dei Sistemi Complessi, Consiglio Nazionale delle Ricerche (CNR), via Madonna del Piano 10, Sesto Fiorentino, Italy I-50019

## 

**Figure 1:**
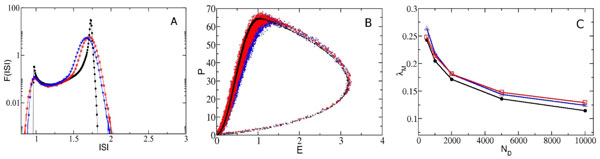
**A. Inter-spike interval distribution functions and **B**. attractor dynamics for a representative case of dilution with system size N_D _= 5000**. **C**. Maximal Lyapunov exponent as a function of network size. In all panels, black circled symbols indicate the diluted deterministic network, blue triangles and red squares denote the stochastic mean-field approximation obtained by employing white and colored noise respectively. For all the panels the deterministic systems are diluted down to the 80%, while the fully coupled stochastic systems are always composed of only 100 neurons.

We consider pulse-coupled leaky integrate-and-fire neural networks with randomly distributed synaptic couplings. This random dilution induces fluctuations in the evolution of the macroscopic variables and deterministic chaos at the microscopic level [1]. Our main aim is to mimic the effect of the dilution as a noise source acting on the dynamics of a globally coupled nonchaotic system. We show that the evolution of a deterministic diluted neural network of any size can be well approximated by a much smaller fully coupled network, where each neuron is driven by a mean synaptic current plus additive noise. These terms represent the average and the fluctuations of the synaptic currents acting on the single neurons in the diluted system. The main microscopic and macroscopic dynamical features can be reproduced within this stochastic approximation. In order to illustrate the quality of this reconstruction, we compare the probability distribution function of the inter-spike intervals and the macroscopic attractor of the deterministic diluted system with those obtained by employing the mean-field stochastic model (see Figure 1A and 1B, respectively). Furthermore, the microscopic stability of the diluted network can be also reproduced, as demonstrated from the almost coincidence of the measured Lyapunov exponents in the deterministic and stochastic cases for an ample range of system sizes (see Figure 1C.). Our results strongly suggest that the fluctuations in the synaptic currents are responsible for the emergence of chaos in this class of pulse-coupled systems [2].
